# Black Americans suppress emotions when prejudice is believed to stem from shared ignorance

**DOI:** 10.3389/fpsyg.2024.1336552

**Published:** 2024-03-18

**Authors:** Kimberly E. Chaney, Minh Duc Pham, Rebecca Cipollina

**Affiliations:** ^1^Department of Psychological Sciences, University of Connecticut, Storrs, CT, United States; ^2^Social Behavioral Sciences, Yale University, New Haven, CT, United States

**Keywords:** racism, expression suppression, lay theories, discrimination, health

## Abstract

Past research examining lay theories of the origins of prejudice has focused on white Americans and has not considered how Black Americans’ lay theories of prejudice may impact emotion regulation following discrimination. Across three samples of Black Americans (*N* = 419), the present research examined relationships between endorsement of two lay theories of prejudice origins (1, beliefs that prejudice stems from shared social ignorance and 2, that prejudice stems from malice). Stronger beliefs that prejudice stems from shared ignorance were associated with greater expression suppression following experiences of racial discrimination (studies 1b and 2), which was, in turn, associated with psychological distress (study 2). By centering the beliefs and experiences of Black Americans in response to discrimination events, the present research has implications for understanding how emotion regulation following racial discrimination is impacted by marginalized groups’ conceptualizations of prejudice. Future research should investigate how these factors impact health disparities.

## Introduction

Despite the growing scientific literature on the causes and implications of anti-Black prejudice (e.g., Pieterse et al., 2012; Yi et al., 2022), less is known about how everyday people think about and understand prejudice. Lay theories are non-experts’ beliefs about the nature of a phenomenon (Anderson and Lindsay, 1998) and function to help make sense of social interactions (Plaks et al., 2005). Research on lay theories of prejudice (LTPs) has explored varied facets of prejudice. For example, research has explored beliefs about common traits among prejudiced people (e.g., being ignorant; Sommers and Norton, 2006), beliefs about how prejudices co-occur (e.g., Chaney et al., 2021), beliefs about the origins of prejudice in others (e.g., Hodson and Esses, 2005), and beliefs about the malleability of prejudice in others (e.g., Neel and Shapiro, 2012, 2015). Critically, research on LTPs can be descriptive: identifying and understanding the content of such lay theories (e.g., Hodson and Esses, 2005). Research moving beyond the description of LTPs documents how LTPs shape attitudes, cognition, and behavior (e.g., Carr et al., 2012; Apfelbaum et al., 2017). However, this research has overwhelmingly focused on White Americans’ endorsement of LTPs and is yet to consider how LTPs may be associated with how marginalized group members cope with discrimination and, consequently, their well-being. In the present research, we focus on Black Americans’ lay theories on the origins of prejudice.

To date, a separate body of literature suggests that experiences of prejudice or discrimination affect marginalized group member’s ability to adaptively regulate their emotions (e.g., Juang et al., 2016; Riley et al., 2021), with adverse health consequences. Following the stress and coping framework (Lazarus and Folkman, 1984), the previous study suggests that accumulative experiences of prejudices, such as racism, place a detrimental toll on one’s psychological and physiological responses to stress (e.g., Burton et al., 2018), which ultimately produce health disparities between marginalized and privileged groups (e.g., Goosby et al., 2018; Smith, 2021). In the present study, we sought to combine the literature on LTPs and stress and coping to examine if Black Americans’ beliefs about prejudice origins are associated with adaptive coping efforts after experiences of racial discrimination.

### Lay theories of prejudice origins

Research has begun to examine the lay theories people hold regarding the origins of prejudice. Among a sample of predominately White U.S. participants, the traits identified as most suiting “racists” were ignorant, close-minded, fearful of change, and hateful (Sommers and Norton, 2006). Other research, while not conducted in the U.S., has more directly assessed beliefs about where prejudices come from. For example, in a predominantly White sample in Canada, the most strongly supported lay theories of ethnic prejudice origins were ignorance (42.0%) and learned from parents (32.7%; Hodson and Esses, 2005). Similar research with Italian high schoolers found comparable beliefs, with ignorance and close-mindedness as the most strongly supported beliefs of prejudice origins (Miglietta et al., 2014).

Critically, past research has considered ignorance and learned theories of prejudice origins as a single factor (e.g., Hodson and Esses, 2005). Indeed, ignorance of LTP suggests that people are unaware of or have yet to learn about cultural differences and systemic inequities. Relatedly, a learned LTP highlights a belief that prejudices are shared and passed on within social communities, reflecting a social learning model of prejudice (e.g., Bigler and Liben, 2007; Miklikowska et al., 2019). Indeed, ignorance about racism (i.e., the tendency to minimize or overlook differences created by race) can be transmitted within families and communities (e.g., Plaut, 2002; Sullivan et al., 2020). Hence, in line with past research (e.g., Hodson and Esses, 2005), we contend that these two beliefs reinforce each other, such that a person may believe prejudice stems from ignorance that is taught and reproduced in social circles. Overall, these beliefs about the origins of prejudice focus on cultures and social environments to explain prejudiced attitudes and thus refer to this belief as *shared ignorance LTP*.

Additionally, participants in prior research have reported a belief that prejudice originates from malice (Sommers and Norton, 2006), a belief that has been constructed as separate from shared ignorance. This lay theory places a heavier individual focus on the origin of prejudice, indicating a belief that prejudice is a result of an affective dislike or hatred. Critically, “hateful” was indicated as highly prototypical, and “cruel” was indicated as moderately prototypical of “White racists” (Sommers and Norton, 2006), suggesting a belief that prejudice stems from malice. However, other research studies have not examined malice as a salient explanation for prejudiced attitudes (Hodson and Esses, 2005; Miglietta et al., 2014). As such, less is known about the belief that prejudice originates in malice. Together, research suggests that White lay people primarily theorize the origins of prejudice as shared ignorance, while a theory that prejudice is a product of hateful beliefs (i.e., malice) was less commonly endorsed. However, little is known about whether such patterns of beliefs generalize to other groups, such as Black Americans.

### Racial discrimination, expression suppression, and psychological distress

Interpersonal and structural racism have been declared a health determinant by the United Nations (United Nations, 2009; Williams et al., 2019). Racism is pervasive (Banaji et al., 2021) and places a biopsychosocial toll on the lives of racial minorities (Clark et al., 1999; Pascoe and Smart Richman, 2009; Paradies et al., 2015). For instance, Black Americans are more likely to have comorbid health conditions and early mortality than White Americans (Pascoe and Smart Richman, 2009; Paradies et al., 2015), and this disparity is exacerbated among Black Americans who live in settings with higher anti-Black racism (e.g., Bailey et al., 2017). Moreover, Black Americans’ psychological health has been attributed to experiences of discrimination (Brown et al., 2000), to the extent that even witnessing anti-Black racism against others is associated with poor mental health outcomes (Bowen-Reid and Tulloch, 2021; Sosoo et al., 2022).

When contending with interpersonal experiences of racism, individuals need to decide how to react to the situation. Some choose to verbally confront prejudice (Czopp et al., 2006; Chaney et al., 2015), while others may let the instance slide and react to the event later, perhaps after seeking advice from others (Chaney and Sanchez, 2022). Such differences in reactions to prejudice often stem from constraints of the social situation, such that confronting may result in backlash for Black confronters (Czopp and Monteith, 2003; Alt et al., 2019), making suppressing emotions seem like an interpersonally safer way to respond. Relatedly, Black Americans, especially Black women, may feel the need to conceal emotional responses to discrimination (e.g., Woods-Giscombé, 2010; Abrams et al., 2019). Indeed, Black people may suppress emotions because they believe displaying emotional reactions gives power to the perpetrator or may elicit unwanted attention from others (e.g., Contrada et al., 2000; Allen et al., 2019).

Emotion suppression (i.e., inhibiting an emotional response while emotionally stimulated; Gross and Levenson, 1997) is a common emotion-regulation response for Black people (Wilson and Gentzler, 2021), perhaps particularly among those who encounter greater prejudice in their daily lives (Lozada et al., 2022; Martinez et al., 2022). However, suppressing one’s emotional reactions to stressors is a maladaptive emotion-regulation strategy. Those who more frequently suppress their emotions from others are more likely to have psychiatric conditions (e.g., Wilson and Gentzler, 2021). Indeed, while previous studies identified expression suppression as a product of experiencing stigma (Hatzenbuehler et al., 2009; Burton et al., 2018), to date, no work has examined the health consequences of suppressing one’s emotions specifically in the context of experiencing racial discrimination. A related study has, however, found that coping with discrimination by detaching (e.g., “I do not talk with others about my feelings,” Wei et al., 2010) is related to poorer psychological health, suggesting that Black Americans who suppress their emotions in response to discrimination may experience psychological distress.

### Current research

While past research has found that LTPs can affect when marginalized people anticipate facing stigma, including impacting cardiovascular reactivity (e.g., Sanchez et al., 2018; Chaney et al., 2021), past research has not examined how lay beliefs about prejudice origins may contribute to Black Americans’ tendencies to suppress emotions. In the present study, we present the first-time measurement of Black Americans’ endorsement of LTP origins (extending previous studies with White samples; see Hodson and Esses, 2005; Sommers and Norton, 2006). As the first examination of Black Americans’ endorsement of LTP origins, we first sought to demonstrate the extent to which Black Americans endorsed different lay theories about prejudice origins (Study 1a). We expected that Black Americans might endorse ignorance LTP and learned LTP more strongly than malice LTP for the following several reasons: (1) colorblindness is a major narrative in understanding U.S. racism (Plaut, 2002); (2) microaggressions and subtle forms of discrimination (which may be deemed as primarily rooted in shared ignorance) are commonplace (Sue et al., 2007); (3) seeing prejudices as more severe such as coming from malice is a potentially harmful coping strategy (Eccleston and Major, 2006); and (4) the past research has documented greater endorsements of ignorance and learned LTPs in White samples (e.g., Hodson and Esses, 2005; Sommers and Norton, 2006). We also examined how these lay beliefs were associated with participants’ frequency of experiencing discrimination (Study 1b), and how these LTPs were associated with emotional suppression and psychological distress measures (Study 1b).

We suggest that beliefs about prejudice origins may be critical in shaping how Black Americans respond to racial discrimination. Particularly, as a belief that prejudice stems from shared ignorance reflects a belief that prejudice is widespread and due to lack of awareness, we hypothesized that a shared ignorance lay belief would be related to greater expression suppression so as not to let prejudice be a bother (e.g., remain resilient). Conversely, we had no hypotheses regarding the effect of malice LTP on expression suppression. That is, malice LTP may make discrimination seem an active, intentional choice, which may make Black individuals feel either more or less motivated to suppress emotional reactions to discrimination.

Study 2 sought to examine, for the first time, how LTP origins impacted Black Americans’ emotion suppression following racial discrimination, employing an experience sampling method with Black American undergraduates (Study 2). This study design expands prior work examining correlations between general emotion suppression and psychological distress related to discrimination experiences (e.g., Wilson and Gentzler, 2021). We hypothesized that LTP origins assessed at baseline would be associated with expression suppression after discrimination events, which in turn would be associated with greater self-reported psychological distress. Finally, we include examinations of relationships with previously identified covariates of reporting and coping with discrimination (e.g., hypervigilance to racial discrimination and racial identity centrality) to demonstrate the unique predictive utility of LTP origins on emotion regulation after racial discrimination. All research was conducted with IRB approval. Data and materials are available at https://osf.io/uqxpc/?view_only=92f432b3f73c444f9105b43b13808af1.

### Study 1a

Study 1 examined LTP endorsement among Black U.S. undergraduates. We explored differences in the endorsement of each of the three origins, alongside the strength of associations between these origins and participants’ reported frequency of experiencing discrimination.

### Method

The sample included 218 Black American participants (*M*_age_ = 18.16, *SD* = 0.97) who completed a mass-testing survey during one of two semesters at a northeastern public university in the U.S. The sample included 144 cisgender women, 66 cisgender men, and 8 non-binary participants. Participants received partial course credit for completing the survey.

Among other questions in the mass-testing survey, participants completed four items assessing LTP origin endorsements (three items assessing their belief that prejudice stems from ignorance, is learned, or stems from malice) and one item assessing how frequently they experience discrimination. Items were presented in a random order in the survey. On a scale from 1 (*Strongly disagree*) to 5 (*Strongly agree*), participants completed one item assessing LTP ignorance, “Prejudice comes from a lack of knowledge about other groups,” one item assessing LTP learned, “Those who are prejudiced learned their prejudicial attitudes from others,” and one item assessing LTP malice, “The source of prejudice is hatred.” On a scale from 1 (*Never*) to 5 (*A great deal*), participants indicated how often they “experience discrimination.”

## Results and discussion

Correlations and descriptive statistics (e.g., sample means and standard deviations) are presented in Table 1. All scales met current standards for reliability (e.g., Hajjar, 2018). Pearson’s correlations, employed due to hypothesized linear relationships, revealed that endorsement of ignorance, learned, and malice LTPs was significantly positively correlated. Comparisons of correlations revealed that ignorance and learned LTPs were more positively correlated than ignorance and malice, *z* = 3.39, *p* < 0.001. Only learned LTP was significantly correlated with more reported discrimination experiences.

**Table 1:** Study 1a correlations and descriptive statistics.

	1	2	3	*M* (*SD*)
1. Ignorance LTP				4.24 (0.94)
2. Learned LTP	0.52**			4.18 (0.87)
3. Malice LTP	0.28**	0.31**		3.64 (1.04)
4. Discrimination	0.13	0.18**	0.09	2.68 (0.91)

*p < 0.05; **p < 0.01.

We next assessed if LTP origin beliefs significantly varied in strength of endorsement. As parametric testing is robust for examining Likert scale outcomes (Norman, 2010), a 3-cell repeated-measures ANOVA examining endorsement of ignorance, learned, and malice LTP origins was significant, *F* (2,430) = 29.74, *p* < 0.001, *d* = 1.06. LSD *post hoc* tests revealed that ignorance and learned LTPs did not significantly differ, *p* = 0.404, *d*_rm_ = 0.07, but both were endorsed significantly more than malice, *p* < 0.001, *ds*_rm_ > 0.44. Study 1a thus indicated a stronger endorsement of ignorance and learned LTPs than malice LTPs. Findings indicated a moderate to high correlation between beliefs that prejudice originates from ignorance and that it is learned. Documented correlations between malice and the other two examined origins were significantly weaker, though positive.

Note that analyses remained consistent when LTP-learned and LTP-ignorance responses were averaged to create a composite LTP-shared ignorance measure. Analyses were again conducted employing ANOVAs. LTP-shared ignorance is a multi-item scale in which item responses are averaged together to create a continuous measure, instead of a single-item Likert measure, which reflects a categorical outcome. Notably, multi-item scales are preferable as they allow for a more robust assessment of theoretically complex constructs such as lay theories of prejudice (Bergkvist and Rossiter, 2007). LTP-shared ignorance was significantly positively correlated with LTP-malice, *r* (217) = 0.333, *p* < 0.001. Similarly, a two-cell within-subjects ANOVA revealed that LTP-shared ignorance (*M* = 4.21, *SD* = 0.79) was endorsed more strongly than LTP-malice (*M* = 3.64, *SD* = 1.04), *F* (1,216) = 61.51, *p* < 0.001, *d*_rm_ = 1.07.

### Study 1b

Having identified endorsement of LTP origins in Study 1a, Study 1b afforded an examination of how LTP origins impact coping following Black Americans’ racial discrimination experiences. Given the high correlation between ignorance and learned LTPs in Study 1a and past research collapsing ignorance and learned LTPs (Hodson and Esses, 2005), we combined these beliefs in the following studies. That is, while the belief that prejudice stems from ignorance suggests prejudices stem from colorblindness, a belief that prejudice is learned suggests that prejudice may be passed on within social groups. We contend that such colorblindness may be considered part of how prejudice is learned from others. We refer to this combined LTP as *shared ignorance* hereon.

Study 1b employed a correlational design to examine how LTP origins (i.e., shared ignorance and malice) are related to how participants recalled responding to racial discrimination. Specifically, we examined relationships between LTP origins and expression suppression after experiencing discrimination, as well as how these LTP origins were associated with indicators of psychological distress (i.e., self-reported stress, anxiety, and depression). We explored whether the greater endorsement of shared ignorance LTP was related to greater expression suppression following discrimination. Furthermore, we examined whether shared ignorance LTP was associated with more psychological distress through associated increases in expressive suppression. We controlled for discrimination frequency given the link between discrimination and psychological distress (e.g., Pascoe and Smart Richman, 2009; Pham and Borton, 2022).

## Method

### Participants

Black American participants were recruited from MTurk via Cloud Research (Litman et al., 2017) for monetary compensation (USD $1.50, 5-min survey). An *a priori* power analysis for a hierarchical regression with two predictors indicated a desired sample size of 130 to detect a small effect (*d* = 0.20) with 90% power. A data collection stop point was set at 150 in case of exclusions. Overall, 150 participants completed the survey, but 5 participants were excluded for failing all instructional attention checks, and 4 were excluded for not identifying as Black in the survey, resulting in an analytic sample of 141 (*M*_age_ = 37.98, *SD* = 12.02). The sample included 88 cisgender women, 50 cisgender men, 1 transgender man, and 2 non-binary participants. About half of the participants indicated working full time (*n* = 76), while 25 indicated part-time work, 15 indicated unemployment, 11 indicated they were retired, 6 were students, 5 were homemakers or stay-at-home parents, and 3 indicated other employment statuses. About half of the sample indicated never being married (*n* = 78), while 35 were married, 17 were living with a partner, and 11 were divorced/separated. A sensitivity power analysis indicated the sample could detect a small effect (*d* = 0.11) with 80% power in the hierarchical linear regression.

## Procedure and materials

Upon providing consent, participants indicated their LTPs, completed measures assessing their discrimination experiences, emotion regulation following discrimination, and an assessment of general wellbeing.^1^ Finally, participants completed the demographics and were debriefed.

### LTPs

Participants completed six items assessing shared ignorance LTP on a scale from 1 (S*trongly disagree*) to 7 (*Strongly agree*). Sample items included “Prejudice comes from a lack of knowledge about other groups” and “People become prejudiced because they aren’t educated about other groups.” Participants completed a three-item measure assessing their belief that prejudice stems from *malice* on the same scale (e.g., “Prejudice results from malice,” “The source of prejudice is hatred of others).” See Table 2 for reliabilities and descriptive statistics.

**Table 2:** Study 1b correlations and descriptive statistics.

	1	2	3	4	5	6	*M* (*SD*)	α
1. Shared ignorance LTP							5.91 (0.95)	0.89
2. Malice LTP	0.18*						5.01 (1.50)	0.92
3. Racial discrimination	−0.03	0.03					2.00 (0.92)	0.94
4. Expression suppression	0.20*	0.06	0.25**				5.06 (1.15)	0.63
5. Stress	−0.08	−0.04	0.55**	0.24**			1.78 (0.71)	0.87
6. Anxiety	−0.09	0.00	0.64**	0.17*	0.76**		1.56 (0.65)	0.88
7. Depression	−0.18	−0.11	0.45**	0.16	0.79**	0.69**	1.74 (0.83)	0.94

*p < 0.05; **p < 0.01.

### Racial discrimination

Expanding on the one-item measure utilized in Study 1a, participants completed a nine-item measure assessing discrimination experiences in the last 4 weeks (Williams et al., 1997). Following the prompt, “Consider the following treatments in relation to your racial and/or ethnic identities. Please indicate how often you experienced them in the past four weeks,” participants were presented with nine items such as, “You are treated with less courtesy than other people” and “You receive poorer service in restaurants or stores.” Participants responded on a scale from 1 (*Never*) to 5 (*A great deal*).

### Expression suppression

Participants completed a three-item measure assessing expression suppression on a scale from 1 (*Very untrue of me*) to 7 (*Very true of me*). Following the prompt, “After I experience prejudice or discrimination,” participants responded to items such as “I try to present an image of strength” and “I keep my problem to myself to prevent from burdening others.” While derived from the superwoman schema scale measuring Black women’s tendency to subscribe to the culturally prescribed superwoman role (e.g., the obligation to present an image of strength and to suppress emotions; Allen et al., 2019), these items reflect an intentional suppression of emotional reactions to discrimination that aligns with emotion suppression not specific to a discrimination event (e.g., Hatzenbuehler et al., 2009).

### Psychological distress

Participants completed indicators of psychological distress that included seven items assessing depression (e.g., I felt that I had nothing to look forward to), seven items assessing anxiety (e.g., I felt I was close to panic), and seven items assessing stress (e.g., I found it difficult to relax; Lovibond and Lovibond, 1995). Following the prompt, “Please read each statement and indicate how much the statement applies to you over the past four weeks,” participants responded to all 21 items on a scale from 1 (*Did not apply to me at all*) to 4 (*Applied to me very much or most of the time*). Items of each subscale were averaged such that higher scores reflect greater levels of depression, anxiety, and stress.

## Results and discussion

While shared ignorance and malice LTP endorsement were positively significantly correlated (Table 2), such as in Study 1a, participants endorsed shared ignorance more strongly than malice, *t* (140) = 6.53, *p* < 0.001, *d*_rm_ = 0.77. Neither LTP nor any of the psychological distress subscales were significantly correlated with discrimination experiences. Shared ignorance was positively correlated with expression suppression: shared ignorance LTP was associated with greater recalled expression suppression in the face of prior racial discrimination.

A two-step hierarchical linear regression was conducted to predict expression suppression. In Step 1, racial discrimination experiences were entered. The model was significant, *R*^2^ = 0.06, *F* (1,139) = 9.07, *p* = 0.003. In Step 2, shared ignorance LTP was entered. Step 2, *R*^2^ = 0.10, *R*^2^Δ = 0.04, *p* = 0.012, accounted for significantly more variance. The full Step 2 model was significant, *F* (2,138) = 7.98, *p* < 0.001. Greater discrimination experiences, *B* = 0.32, *SE* = 0.10, *p* = 0.002, and greater shared ignorance LTP endorsement, *B* = 0.25, *SE* = 0.10, *p* = 0.012, significantly predicted greater expression suppression.^2^ An identical hierarchical linear regression examining malice LTP indicated that Step 2 (wherein malice LTP was entered) did not account for more variance than Step 1, *R*^2^Δ = 0.003, *p* = 0.541. The Step 2 model was significant, *F* (2,138) = 4.70, *p* = 0.011. Malice LTP did not significantly predict expression suppression, *B* = 0.04, *SE* = 0.06, *p* = 0.541, but discrimination experiences predicted expression suppression, *B* = 0.31, *SE* = 0.10, *p* = 0.003.

Three mediation models examining the relationship of shared ignorance LTP on stress, anxiety, and depression via expression suppression, including the discrimination experience as a covariate, indicated that while shared ignorance LTP significantly predicted greater expression suppression following discrimination, *B* = 0.25, *SE* = 0.10, *p* = 0.012, 95% CI [0.06, 0.44], expression suppression did not significantly predict anxiety, *B* = 0.02, *SE* = 0.04, *p* = 0.683, 95% CI [−0.06, 0.09], depression, *B* = 0.07, *SE* = 0.06, *p* = 0.270, 95% CI [−0.05, 0.18], or stress, *B* = 0.08, *SE* = 0.05, *p* = 0.100, 95% CI [−0.01, 0.17]. The indirect effect via expression suppression was not significant for anxiety, *B* = 0.01, *SE* = 0.01, 95% CI _boot_ [−0.02, 0.03]; depression, *B* = 0.02, *SE* = 0.02, 95% CI _boot_ [−0.01, 0.05]; or stress, *B* = 0.02, *SE* = 0.01, 95% CI _boot_ [0.00, 0.05].

Study 1b demonstrated that a shared ignorance LTP is related to greater expression suppression following experiences of racial discrimination for Black Americans. Black Americans endorsed shared ignorance LTP more strongly than malice LTP. Furthermore, shared ignorance LTP was associated with greater expression suppression above the influence of discrimination frequency, although expression suppression was not significantly associated with distress indicators. We anticipate that this null finding is a product of the study recall design (4-week retrospect), which we sought to address with Study 2.

### Study 2

While Study 1b afforded evidence that shared ignorance LTP endorsement is associated with Black Americans’ expression suppression after experiencing racial discrimination, the paradigm relied on recalled emotion regulation following prior discrimination experiences. Study 2 employed an experience sampling design to capture Black Americans’ emotion regulation and psychological distress following a discrimination experience within the last 48 h. In doing so, Study 2 examined expression suppression in reaction to a specific, recent experience of racial discrimination and reports of psychological distress following that discrimination. This experience sampling method is especially notable given that the majority of research examining the associations between discrimination, emotion-regulation strategies, and psychological distress employ cross-sectional designs and require reports of emotion regulation on a general level, not to a specific event (e.g., Gilbert and Zemore, 2016; Andrade et al., 2021). Additionally, assessing LTP endorsement at baseline affords a stronger examination of the directionality of the relationship between LTPs and expression suppression following discrimination.

Furthermore, vigilance to discrimination shapes attributions to discrimination and is related to psychological distress (e.g., Miller and Saucier, 2018; Watson-Singleton et al., 2019), and racial identity centrality can impact how Black Americans cope with discrimination (e.g., Christophe et al., 2022; Haeny et al., 2023). As such, Study 2 included racial identity centrality and vigilance to racial discrimination as covariates to demonstrate the predictive utility of LTPs on emotion regulation. We hypothesized that shared ignorance LTP endorsement would be related to greater expression suppression following discrimination, and expression suppression would be related to more psychological distress. Given the null relationship between malice LTP and expression suppression in Study 1b, we hypothesized no significant relationship in Study 2.

## Method

### Participants

Participants who identified as Black during a large prescreen for an undergraduate participant pool were eligible for the study. Participants were attending a predominately White public university in the northeastern U.S.^3^ Every Black participant in the participant pool during two academic semesters was recruited (*N* = 129); thus, our sample size was based on this constraint. While 129 participants completed the baseline survey, 24 did not complete any of the experience sampling surveys and were excluded. Of those who completed any experience sampling surveys, 33 reported not experiencing racial discrimination, 8 did not identify as Black, and 4 participants failed to complete a full experience sampling survey, leaving an analytic sample of 60 participants (*M*_age_ = 18.77, *SD* = 0.84, range: 18–21) who identified as Black monoracial (*n* = 53) or Black multiracial (*n* = 7). The sample was predominantly cisgender women (*n* = 43; cisgender men = 15, genderqueer = 2), and heterosexual (*n* = 43; bisexual = 7, asexual = 5, lesbian/gay = 1, and other identity = 4). A sensitivity power analysis indicated that the sample could detect a small effect (*d* = 0.27) with 80% power.

## Procedure and materials

Participants were recruited via an undergraduate participant pool in exchange for partial course credit. Participants were able to complete the baseline survey on their own computer at any time. The baseline survey included Study 1b measures of LTP origins and a measure of racial identity centrality and demographics. At the end of the baseline, participants were informed that they would receive a brief survey via an email from the participant pool website every other day for the next 2 weeks, and they would receive additional credit for completing each of the six possible surveys. All participants were asked to complete their surveys within 48 h.

At the beginning of each survey, participants were prompted with questions assessing (1) discrimination vigilance and (2) racial discrimination in the past 48 h. If participants indicated experiencing one or more discrimination events in the last 48 h, they completed the measure of expression suppression (Study 1b), followed by an abbreviated measure of psychological distress (as described below). Finally, participants were provided with a text box to describe the discrimination experience, although only 35% of participants opted to describe it. At the end of the 2 weeks, participants received a debriefing message and full compensation of course credit.

### Baseline race centrality

Participants completed a four-item measure of racial identity centrality (Luhtanen and Crocker, 1992) on a scale from 1 (*Strongly disagree*) to 7 (*Strongly agree*). Sample items include, “In general, belonging to my racial group(s) is an important part of my self-image” and “The racial group(s) I belong to are an important reflection of what kind of a person I am.” See Table 3 for reliabilities, correlations, and descriptive statistics.

**Table 3:** Study 2 correlations, descriptive statistics, and scale reliability.

	1	2	3	4	5	6	*M* (*SD*)	*α*
1. Shared ignorance LTP							5.89 (1.07)	0.91
2. Malice LTP	0.36**						4.99 (0.97)	0.78
3. Race centrality	0.38**	0.28*					5.21 (1.08)	0.67
4. Discrimination exp.	−0.13	0.03	−0.05				5.67 (2.35)	-
5. Vigilance	−0.21	0.09	−0.03	0.50**			4.30 (2.23)	-
6. Expression suppression	0.10	0.11	−0.22	0.41**	0.40**		3.85 (1.61)	0.73
7. Psychological distress	0.14	−0.15	−0.06	0.31*	0.28**	0.51**	1.90 (0.61)	0.86

*p < 0.05; **p < 0.01.

### Vigilance

At the beginning of each survey, participants responded on a scale from 0 to 10+ times to the item: “In the past 48 h, how often have you adjusted your behavior to avoid facing racial or ethnic discrimination? This could include, for example, adjusting or thinking about how your appearance could be viewed by others, watching what you say or how you say it, being vigilant to what happens around you, or avoiding certain social situations and places.”

### Discrimination experiences

After the measure of vigilance, participants were asked, “In the past 48 h, how often would you say you experienced racial or ethnic discrimination? This could include being treated with less courtesy or respect than others, people inferring something about you based on racial stereotypes or any other form of blatant discrimination or racial microaggression.” Participants again responded on a scale from 0 to 10+ times.

### Psychological distress

Only participants who indicated experiencing discrimination in the last 48 h completed an abbreviated version of Study 1b’s measure of psychological distress. The nine-item measure included the prompt, “Please read each statement and indicate how much the statement applies to you the past 48 h.” Participants completed three items assessing depression, three assessing anxiety, and three assessing stress. These items were selected as the highest-loading items from Study 1b. Due to low variability, these items were averaged to create an overall psychological distress measure.

## Results and discussion

Analyses were conducted on only the first survey in which participants indicated experiencing discrimination. For the majority of the analytic sample, this was within one of the first three received surveys (Survey 1 = 19, Survey 2 = 17, Survey 3 = 15, Survey 4 = 4, Survey 5 = 5, and Survey 6 = 0) resulting in the discrimination experience being captured, on average, 4.83 days after baseline (*SD* = 2.76). Notably, 33.33% of participants indicated experiencing discrimination in only one survey, 33.33% reported experiencing discrimination in two surveys, 15% reported experiencing discrimination in three surveys, 15% reported experiencing discrimination in four surveys, and 3.3% reported experiencing discrimination in five surveys. This low within-person variability prohibited an examination of expression suppression and psychological distress over time. Replicating Study 1b, a paired-sample *t*-test revealed greater shared ignorance LTP endorsement than malice LTP, *t* (58) = 5.91, *p* < 0.001, *d*_rm_ = 0.74. Note that 98% of the qualitative data describing the instance of discrimination reflected more subtle forms of discrimination than overtly hostile experiences (as coded by two independent coders).

A two-step hierarchical linear regression was conducted to predict expression suppression. In Step 1, racial centrality, discrimination experiences, vigilance, and time (in days) between T1 and discrimination experience were entered. In Step 2, shared ignorance LTP was entered. Step 2, *R*^2^Δ = 0.08, *p* = 0.013, accounted for significantly more variance. The Step 2 model, presented in Table 4, was significant, *F* (5,54) = 5.56, *p* < 0.001. While greater race centrality was related to less expression suppression, discrimination experiences, vigilance, and shared ignorance, LTP endorsement was related to more expression suppression following discrimination.

**Table 4:** Study 2 hierarchical regression predicting expression suppression.

Step 2	*B* (*SE*)	*t*	*p*
Race centrality	−0.47 (0.18)	−2.65	0.011
Vigilance	0.22 (0.09)	2.47	0.017
Discrimination exp.	0.20 (0.09)	2.14	0.037
Time between surveys	0.01 (0.07)	0.08	0.937
Shared ignorance LTP	0.48 (0.19)	2.58	0.013

An identical hierarchical linear regression examining the addition of malice LTP at Step 2 indicated that Step 2 did not account for significantly more variance, *R*^2^Δ = 0.02, *p* = 0.227. The full Step 2 model was significant, *F* (5,53) = 4.69, *p* = 0.001, but malice LTP was not significantly related to expression suppression after discrimination, *B* = 0.24, *SE* = 0.20, *p* = 0.227.^4^

**Figure 1:**
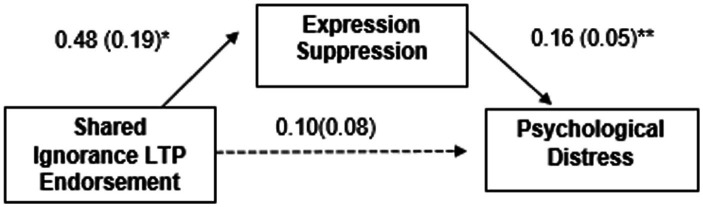
Study 2 mediation. Unstandardized regression coefficients and standardized errors are presented. *p < 0.05, **p < 0.01.

A mediation model examining the effect of shared ignorance on psychological distress via expression suppression, including covariates of the above model, indicated that shared ignorance significantly predicted greater expression suppression following discrimination, and expression suppression following discrimination was related to greater reported psychological distress (see Figure 1). While the direct effect of shared ignorance on psychological distress was not significant, *B* = 0.10, *SE* = 0.08, 95% CI [−0.06, 0.25], the indirect effect via expression suppression was significant, *B* = 0.08, *SE* = 0.04, 95% CI _boot_ [0.01, 0.16].^5^

Study 2 demonstrated that, when accounting for racial identity centrality, vigilance, and broader discrimination experiences, greater shared ignorance LTP was related to greater expression suppression following discrimination and, in turn, greater psychological distress. In contrast, malice LTP was unrelated to expression suppression following discrimination experience. By assessing expression suppression and psychological distress within 48 h of discrimination and assessing LTP endorsement during a prior baseline, the present research offers evidence of the effect of shared ignorance of LTP endorsement on coping.

It is noted that while shared ignorance LTP was directly and significantly related to expression suppression after discrimination experiences broadly with an online sample of Black Americans in Study 1b, this direct relationship was not significant in Study 2 when assessing expression suppression following a specific incident of discrimination among Black college students. Rather, in Study 2, when accounting for Black college students’ broader experiences of discrimination and their racial identity, a significant relationship between shared ignorance LTP and expression suppression emerged. Such an effect may suggest that expression suppression may be most likely to emerge among Black Americans who experience discrimination, and are more vigilant to discrimination, over time.

## General discussion

Research on LTP origins has primarily focused on White American’s endorsement (Hodson and Esses, 2005; Sommers and Norton, 2006) and has not considered that LTPs may impact how Black Americans cope with racial discrimination. The present studies demonstrate, for the first time, that Black Americans more strongly endorse a belief that prejudice stems from shared ignorance than from malice (Studies 1 and 2). Moreover, shared ignorance endorsement was associated with greater expression suppression following racial discrimination among Black adults recruited online (Study 1b) and from an undergraduate sample (Study 2). This research examined, for the first time, expression suppression in response to a racist incident and indicated that expression suppression was associated with psychological distress outcomes following racial discrimination for Black undergraduate students (Study 2).

The identified relationship between discrimination-specific expression suppression and psychological distress following a recent racist encounter is novel to the literature in multiple ways. First, while previous studies have demonstrated that the frequency of experiencing discrimination is associated with the general frequency of expressing emotions (Wilson and Gentzler, 2021; Lozada et al., 2022; Martinez et al., 2022), these findings lack clarity on whether expression suppression was utilized in response to discrimination experiences. Second, the expression suppression measure utilized in this study extends a previous study on emotion-regulation strategies that are employed among individuals reacting to racism (e.g., Wei et al., 2010). Finally, the demonstrated negative impact of expression suppression speaks to the insidious nature of strength-related stereotypes of Black Americans: perceived high pain tolerance associated with Black Americans (Hoffman et al., 2016), the societal pressure to be hypermasculine among Black men (Young, 2021), and the superwoman schema among Black women (i.e., Black women trying to fit a “superwoman” ideal of not displaying or burdening others with their emotions; Woods-Giscombé, 2010; Allen et al., 2019). Notably, as our measure of expression suppression closely resembles items utilized in prior work on the superwoman schema, current findings suggest that this schema may be related to increased emotional suppression in the face of racial discrimination. However, the present studies were not statistically powered to test for gender effects; thus, we encourage future research to examine potential gender differences or how this schema may impact Black Americans more broadly.

### Implications

The present research highlights two LTP origins endorsed by Black Americans and their relationships with responses to discrimination. Specifically, a shared ignorance belief, but not a malice belief, may contribute to expression suppression. We contend that Black Americans tend to feel the need to “shoulder” the struggle and remain resilient more when believing prejudice stems from shared ignorance because then, prejudice seems more passive, and it is more difficult to locate its cause. In comparison, perceiving prejudice as stemming from malice, or as an active, intentional act, may make Black people more or less likely to suppress their emotions depending on various individuals (e.g., confrontation style and perceived effectiveness of confronting perpetrator; Chaney & Wedell, 2022) and situational (e.g., prejudice type and perpetrator identity) factors. We caution, however, against an interpretation of our findings that it would be better for people to *not* endorse shared ignorance LTP or that people should endorse malice LTP. Rather, the present research demonstrates an additional contributor to expression suppression following racial discrimination. By better understanding the varied factors that lead people to engage in expression suppression, research can better address the motives for this emotion-regulation strategy.

Furthermore, we contend that efforts to better understand how Black Americans conceptualize prejudice are integral in advancing racial health equity research, as such conceptualizations may be pivotal in shaping how Black Americans cope with discrimination. The present research affords a person-centered approach that centers Black Americans’ beliefs (i.e., LTPs) in the study of coping with discrimination. This research establishes a theoretically novel advancement to understanding the impact of discrimination on Black Americans’ health, and it provides greater insights into the individual differences (i.e., LTP endorsements) that shape experiences of and reactions to anti-Black discrimination. The identification of LTPs endorsed by Black Americans in the present samples and evidence of LTP health correlates affords a foundation for future approaches to health disparities research by centering the beliefs of Black Americans. For instance, a recent qualitative study has tested the efficacy of an intervention to improve Black women’s use of adaptive emotion-regulation strategies after discrimination (Conway-Phillips et al., 2020), and it may be advanced by integrating research on LTPs.

Finally, current findings reveal that lay people recognize the role of shared ignorance in the development and spread of racism (a relationship well documented in the scientific literature, e.g., Bonilla-Silva, 2006). We could notice the ironic effect that a felt need to suppress expressions in response to discrimination because expressing emotions or further engaging in the incident could lead to worse outcomes such as backlash at the time of the racist incident (Contrada et al., 2000; Allen et al., 2019); yet, expression suppression ultimately contributes to poorer wellbeing. Hence, while it may be beneficial for Black individuals to adaptively regulate emotions, research must avoid pathologizing and placing the responsibility on Black people (Johnson, 2022).

### Future directions

Notably, by examining Black American’s LTPs from previous studies on White samples (Hodson & Esses, 2005; Sommers & Norton, 2006), we acknowledge that our approach is Eurocentric and encourage future research to adopt a qualitative approach to assess any novel LTPs held by Black Americans. Moreover, shared ignorance and malice LTPs were positively correlated (*r*s: 0.18–0.36), suggesting such beliefs are not in opposition. LTPs need not be singularly endorsed. While LTPs represent general beliefs, such beliefs can be flexible in response to varied situations and targets (Neel and Lassetter, 2015; Chaney and Chasteen, 2023). For example, people may view some discrimination as originating from one source (e.g., hate crimes may be viewed as stemming from malice), while other discrimination forms as originating from a separate source (e.g., microaggressions may be viewed as stemming from shared ignorance). It will be critical for future research to integrate situation- and target-level variabilities.

The present research did not demonstrate a causal effect of shared ignorance on expression suppression as it relied on correlational designs. Future research could manipulate LTPs (Carr et al., 2012; Chaney & Forbes, 2023) to demonstrate causal changes in emotion regulation following discrimination. Moreover, while Study 2 utilized an experience sampling design across the 2-week study period, we were unable to examine within-participant variance in emotion regulation due to few participants reporting frequent experiences of discrimination. However, it will be critical for research to examine the compounding effects of frequent expression suppression following discrimination experiences. Furthermore, Studies 1a and 2 relied on samples of Black undergraduates at a primarily White institution (PWI). As students of color attending PWIs may experience more discrimination due to their increased interactions with White peers (e.g., Camacho & Quinn, 2023), we encourage future research to recruit more diverse community samples of Black Americans and consider neighborhood, college, or workplace racial diversity when examining the effects of LTPs on expression suppression.

Furthermore, future work should consider identity factors that may be associated with LTP endorsement and expression suppression in the face of discrimination. For instance, as LTPs may be learned and taught within families, future work can explore how parental racial socialization (see Dunbar et al., 2017) can protect Black youth from maladaptive emotion-regulation strategies. Additionally, although participant demographics had no effect on expression suppression or LTPs in the present study (see Supplementary Material), different identity dimensions should be examined in future research (Seng et al., 2012; Pham et al., 2023). Finally, the present research focused on Black Americans’ perceptions of the origins of “prejudice,” not “racism” or “anti-Black racism.” While we hypothesize that “racism” might be the most salient form of prejudice for Black Americans, we encourage future research to consider whether LTP origins may vary when racism is highlighted, rather than prejudice broadly. Indeed, racism may be understood more broadly as both biased interpersonal behaviors and oppressive structures/systems (e.g., Salter et al., 2018; Pham et al., 2023), thus resulting in different patterns of LTPs.

## Conclusion

Employing cross-sectional and micro-longitudinal (i.e., experience sampling) designs, the present study demonstrates that believing prejudice originates in shared ignorance is related to greater expression suppression in response to racial discrimination among Black Americans, particularly when accounting for broader experiences of discrimination, which is in turn associated with greater psychological distress. These findings highlight the understudied role of LTPs on Black Americans’ wellbeing and identify implications for future studies on coping with racial discrimination and adaptive emotion regulation in the discrimination context.

## Data availability statement

Data is openly available at: https://osf.io/uqxpc/?view_only=92f432b3f73c444f9105b43b13808af1.

## Ethics statement

The studies involving humans were approved by the UConn Institutional Review Board. The studies were conducted in accordance with the local legislation and institutional requirements. The participants provided their written informed consent to participate in this study.

## Author contributions

KC: Conceptualization, Formal analysis, Methodology, Writing – original draft. MP: Conceptualization, Methodology, Project administration, Writing – review & editing. RC: Conceptualization, Formal analysis, Writing – review & editing.

## Footnotes

^1^A measure of vigilance to discrimination was assessed and is included in the Supplementary Material as it was exploratory.

^2^Shared ignorance remained a significant predictor of expression suppression when controlling for demographics and vigilance (as presented in the Supplementary Material). The identified pattern of results remains similar when analyses examine ignorance LTP and learned LTP separately (see Supplementary Material). Furthermore, when shared ignorance and malice LTP are entered in Step 2 simultaneously, effects do not significantly change.

^3^An undergraduate sample was selected due to logistical constraints.

^4^The model remains significant if shared ignorance and malice are entered simultaneously. Shared ignorance, *B* = 0.44, *SE* = 0.30, *p* = 0.027, but not malice, *B* = 0.08, *SE* = 0.20, *p* = 0.702, predicts expression suppression.

^5^Results do not significantly change when gender and sexual orientation are entered as covariates, as presented in the Supplementary Material. Results examining ignorance and learned LTP separately are reported in the Supplementary Material.
