# Supplementary material for: Black Americans suppress emotions when prejudice is believed to stem from shared ignorance
Source: Front Psychol. 2024 Mar 18;15:1336552. doi: 10.3389/fpsyg.2024.1336552 (PMC10982414; doi:10.3389/fpsyg.2024.1336552)
Supplement: Supplementary file 1 [file Table_1.DOCX]

**SUPPLEMENT**

**Study 1b**

**Examining learned and ignorance as separate LTPs**

Hierarchical linear regression models identical to the one reported in the manuscript were conducted examining ignorance and learned separately. While ignorance significantly predicted expression suppression, *B* = 0.24, *SE* = 0.08, *p* = .002, learned did not, *B* = 0.14, *SE* = 0.10, *p* = .188.

**Hierarchical linear regressions with demographic covariates**

A hierarchical linear regression model predicting expression suppression that included demographics age, gender, sexual orientation, marital status in Step 1, (Gender was coded as 1= cisgender woman, 2 = cisgender man, 3 = non-binary or another identity. Sexual orientation was coded as 0 = heterosexual, 1 = sexual minority, and marital status was coded as 1 = married, 0 = never married, divorced, or widowed), discrimination experiences and vigilance to discrimination in Step 2, and shared ignorance in Step 3. Step 2, *R*^2^Δ = 0.10, *p* < .001, and Step 3, *R*^2^Δ = 0.03, *p* = .047, accounted for significantly more variance. The full Step 3 model was significant, *F*(7,133) = 3.58, *p* = .001, and is presented in Supplemental Table 1.

**Supplemental Table 1**

*Hierarchical linear regression predicting expression suppression, Study 1b*

|  | *B* (*SE*) | *p* |
| --- | --- | --- |
| Gender | 0.10 (0.18) | .585 |
| Sexual Orientation | -0.01 (0.27) | .973 |
| Marital Status | -0.28 (0.22) | .205 |
| Age | 0.00 (0.09) | .896 |
| Vigilance | 0.27 (0.12) | .025 |
| Discrimination | 0.18 (0.12) | .146 |
| Shared Ignorance | 0.20 (0.10) | .047 |

**Study 2**

**Examining learned and ignorance as separate LTPs**

The indirect effect of individual ignorance LTP on mental health via expression suppression controlling for the covariates reported in the manuscript remains significant, *B* = 0.08, *SE* = 0.04, 95% CI_boot_ [0.01, 0.16].

The indirect effect of individual learned LTP on mental health via expression suppression controlling for the covariates reported in the manuscript is not significant, *B* = 0.05, *SE* = 0.04, 95% CI_boot_ [-0.02, 0.13].

**Mediation analysis controlling for demographic variables**

The indirect effect of shared ignorance on mental health via expression suppression remains significant when controlling for gender and sexual orientation, *B* = 0.08, *SE* = 0.04, 95% CI_boot_ [0.001, 0.16] (Gender was coded as 1 = cisgender woman, 2 = cisgender man, 3 = another gender identity; Sexual orientation was coded as 0 = heterosexual, 1 = sexual minority).
